# Effect of Platform Type on Clinical Efficacy of SARS-CoV-2 Vaccines in Prime Vaccination Settings: A Systematic Review and Meta-Regression of Randomized Controlled Trials

**DOI:** 10.3390/vaccines12020130

**Published:** 2024-01-26

**Authors:** Sergey Goryaynov, Olesya Gurova

**Affiliations:** 1 Independent Researcher, 109544 Moscow, Russia; 2 Department of Endocrinology No. 1, N.V. Sklifosovsky Institute of Clinical Medicine, Sechenov First Moscow State Medical University, 119435 Moscow, Russia; gurova79@inbox.ru

**Keywords:** coronavirus, SARS-CoV-2, COVID-19, vaccine, meta-analysis

## Abstract

This systematic review investigated the association between platform type and the clinical efficacy of SARS-CoV-2 vaccines using the meta-regression of randomized controlled trials to compare the rates of the first appearance of symptomatic COVID-19 on the platforms. The trial search was conducted using PubMed, ClinicalTrials.gov, and the EU Clinical Trials Register. The main selection criteria included: non-active control, immunocompetent individuals without previous vaccination, and a low risk of bias. The platform effect was summarized with an incidence rate ratio (IRR) and a 95% confidence interval for every platform category against the reference. IRR was obtained by random-effect meta-regression with adjustment for confounding by effect modifiers. The analysis was conducted in per-protocol (PP) and modified intention-to-treat (mITT) sets. Six vaccine types with 35 trials were included. Vector vaccines were a reference category. In the PP set, rates of symptomatic COVID-19 on mRNA and protein subunit vaccines were significantly lower than on the vector: IRR = 0.30 [0.19; 0.46], *p* = 0.001 and 0.63 [0.46; 0.86], *p* = 0.012, respectively. There was no difference for inactivated and virus-like particle vaccines compared to the vector: IRR = 0.98 [0.71; 1.36], *p* = 0.913 and 0.70 [0.41; 1.20], *p* = 0.197, respectively. The rate of cases on DNA vaccines was significantly higher than that on the vector: IRR = 2.58 [1.17; 5.68], *p* = 0.034. Results for the mITT set were consistent. Platform type is an effect modifier of the clinical efficacy of SARS-CoV-2 vaccines.

## 1. Introduction

The pandemic of severe acute respiratory syndrome coronavirus 2 (SARS-CoV-2) and the diverse nature of the disease caused by it (COVID-19) showed that communicable diseases are still capable of introducing a healthcare thereat exceeding that of chronic conditions. SARS-CoV-2 provided a powerful boost to the vaccine industry, and vaccines with different platform types were introduced in a short time. However, clinical trials showed variable vaccine efficacy, raising a question about the reasons for such diversity [1]. One explanation can be the presence of intrinsic differences between the platforms, affecting vaccine efficacy. This hypothesis has important implications for healthcare, as it allows for a prioritization of vaccine types with the greatest potential. Evidence for inference on this is lacking: there are no head-to-head clinical comparisons of platforms, and ongoing observational studies focus on immunological endpoints [2]. The latter point is additionally challenged by the lack of strong immunological surrogates of the clinical efficacy of the vaccines [3]. Nevertheless, available clinical trials of SARS-CoV-2 vaccines provide a large amount of high-quality empirical data for analysis that is able to shed light on the comparative efficacy of different platform types. There are no studies specifically designed for this purpose. We conducted a systematic review and meta-regression to investigate associations between platform type and the clinical efficacy of SARS-CoV-2 vaccines. The study objective was to compare rates of the first cases of symptomatic COVID-19 between SARS-CoV-2 vaccine platforms in immunocompetent individuals who are indicated but were not previously exposed to SARS-CoV-2 vaccine, using the results of available randomized controlled trials (RCT).

## 2. Methods

### 2.1. Data Sources and Search

After finalization of the protocol, the study was registered in PROSPERO with CRD42023447481. Only databases with free access were used in the search for trials in this study. The search was conducted in three databases: PubMed, ClinicalTrials.gov, and the EU Clinical Trials Register. The basic search request was “(SARS-CoV-2 OR COVID-19 OR coronavirus) AND (vaccine OR vaccination OR prevention)”. The search in PubMed was restricted to randomized controlled trials. The search in ClinicalTrials.gov was restricted to interventional studies with completed recruitment. There were no other restrictions (date, language, publication format, etc.). Searches were conducted in all databases simultaneously, with the results downloaded. Downloaded versions of the search results were used for all further activities related to the identification of trials for the study. For consistency of searches across the databases, different records and publications related to the same trial were considered as single. One author (S.G.) performed the search, removed inter- and between-database duplicates, and prepared lists of unique records for each database, which were used during the rest of the review.

### 2.2. Study Selection

Only trials meeting all the following criteria were eligible: parallel group or factorial design, SARS-CoV-2 vaccine as an intervention, non-active control, no background therapy, immunocompetent individuals, no prior SARS-CoV-2 vaccination, investigation of vaccine effect on COVID-19 occurrence listed in trial objectives, use of reverse transcription polymerase chain reaction (RT-PCR) for COVID-19 cases confirmation, proper randomization, and blinded COVID-19 endpoint assessment. Criteria of proper randomization were the use of sequence generation and concealment methods, supported by baseline group characteristics, in line with requirements of RoB 2.0 for low risk of bias arising from the randomization process [4]. Initially, eligibility criteria also comprised a threshold of ≥50% for vaccine efficacy. The aim was to separate vaccines with insufficient immunogenicity, and thus inappropriate for clinical use, and to remove another source of heterogeneity in the trial results. After the start of the study, this criterion was found to be impractical to assess and incompatible with the variable SARS-CoV-2 environment, resulting in subjective interpretation and the risk of introducing selection bias. On this basis, the protocol was amended with the efficacy threshold excluded and not used in the study.

Both authors independently screened, assessed eligibility, and prepared lists of candidates for inclusion in the study. Candidate lists were compared and merged to form the final list of trials meeting the eligibility criteria. Inconsistencies were resolved by consensus.

### 2.3. Endpoint

The only endpoint in the study was the incidence of first cases of symptomatic COVID-19 of any severity confirmed by positive results of RT-PCR for SARS-CoV-2.

### 2.4. Data and Extraction, Quality Assessment

Double-data extraction was used. Both authors independently extracted all trial data, followed by cross-verification and merge, with inconsistencies resolved by consensus. All data items were collected with and stored in the dedicated Excel-based data collection form. Data were manually copied from their sources with the location recorded for every data item. Different reports of the same trial were prioritized as follows: later analysis over earlier, analysis with larger number of events/observations, and adjusted analysis over unadjusted. Only publicly available sources with free access were used to collect trial data. No direct contacts with trial authors were made. In the absence of information on any extracted data item in the records identified during the primary search, an additional non-systematic web-search was conducted.

Types of the extracted data were: trial identifiers; references used as data sources; dates of enrollment and data cut-off; characteristics related to trial design, eligibility criteria, vaccine and its dosing regimen, endpoint definition and measurement; definition and baseline characteristics of analysis populations; group-level and summary statistics for the endpoint; data related to effect modifiers of vaccine efficacy; and characteristics related to the trial performance and endpoint measurement.

There was no assessment of bias risk in individual trials as measures to control it were set with the eligibility criteria and data synthesis strategy. The risk of publication bias was assessed with the funnel plot and Egger’s test.

### 2.5. Data Synthesis and Analysis

Eligible trials were grouped and categorized according to the type of vaccine platform used. Vaccine efficacy on the endpoint at the trial-level was summarized with incidence rate ratio (IRR) and 95% confidence interval (CI) (vaccine in numerator, control in denominator). Point and interval estimates of IRR were calculated from vaccine efficacy parameter (VE) in percentages. Heterogeneity of the trial results was estimated with the I^2^ and Q-test. Between-trial variance was estimated using the restricted maximum likelihood (REML) as less biased [5].

The effect of platform type on vaccine efficacy on the endpoint was summarized with IRR and 95% CI for every platform category against the reference (numerator and denominator, respectively) and obtained by meta-regression with the platform type category as a binary covariate. The platform category with the largest total number of participants in the trials was the reference. Meta-regression was done on the log-scale. Statistical testing of covariates was conducted with the z-test. The random-effect model and permutation test were used to control the risk of false-positive results in statistical inferences [6].

Analysis of the platform effect was prespecified to be adjusted for confounding by effect modifiers (vaccine- and population-level) of vaccine efficacy and bias due to trial performance or endpoint measurement. Part of these variables were prespecified in the protocol at the planning stage, and the rest were identified ad hoc after review of the eligible trials. To avoid model overfitting and a decrease in power caused by redundant covariates, a parsimony principle was used for the meta-regression model fitting: the highest goodness-of-fit with the least total number of covariates. Forward stepwise selection of covariates was used for this purpose with only informative ones kept in the model. Goodness-of-fit of the model was assessed with R^2^ analogue.

Trial-level IRR and CI were calculated from rates and events if VE values were missing. Lacking rates were calculated from group-level data or approximated from proportions. Continuity correction was used for trials with zero events in any group [7].

Data synthesis was based on the observed cases and conducted in per-protocol (PP) and modified intention-to-treat (mITT) analysis sets. The PP set included trial results in analysis populations comprising participants who completed full vaccination and were seronegative for SARS-CoV-2 before the start of the COVID-19 cases count for the endpoint. The mITT set included trial results in analysis populations with participants who received at least one dose of vaccine, regardless of serologic status, with the COVID-19 cases count starting after the first dose.

The dataset was prepared in MS Excel. Data synthesis was conducted in R (version 4.0.5) with RStudio interface (version 2023.06.2 build 561).

## 3. Results

### 3.1. Search

**Figure 1:**
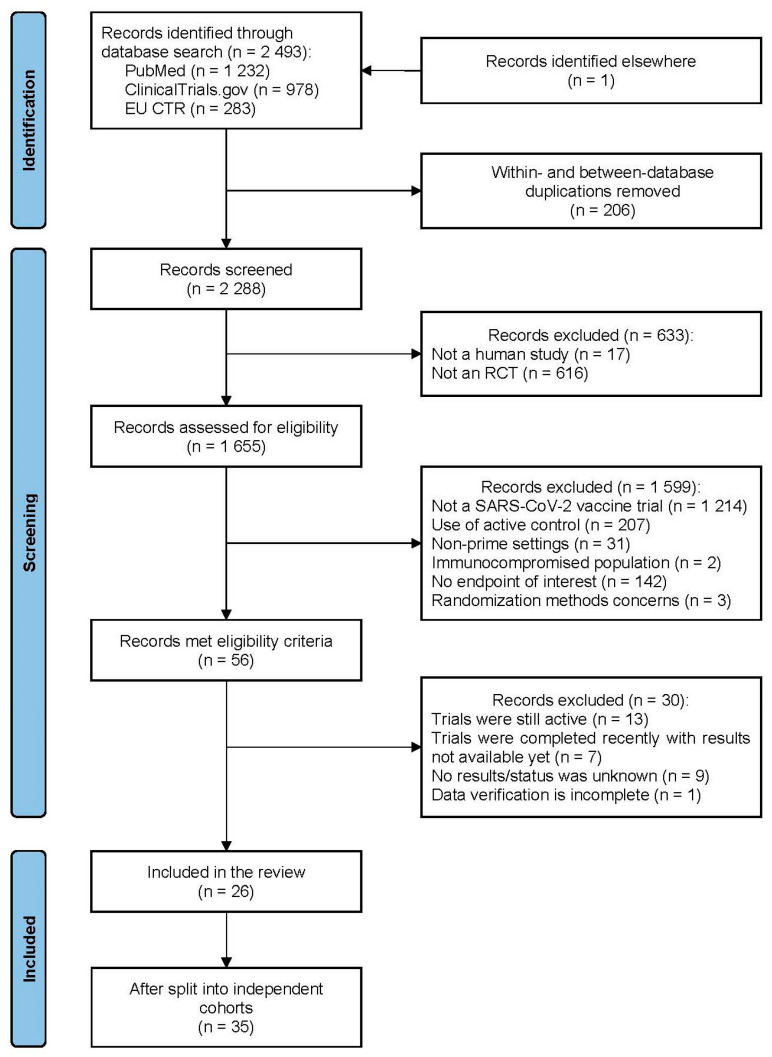
PRISMA diagram for the study.

**Figure 2:**
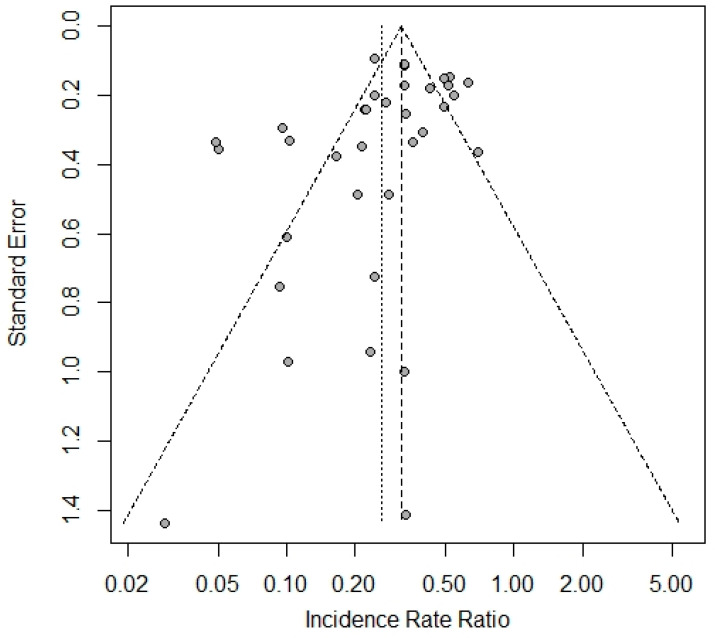
Funnel plot for the included trials.

The overall process of the trial identification and selection is presented in Figure 1. The search results cutoff was completed on 7 August 2023. One fully eligible trial not indexed by the primary search was accidentally identified later during the search of data for other trials. One fully eligible trial was not included due to its incomplete data verification. Part of the included studies were clinical programs that comprised several independent cohorts aggregated under the same acronym/registration number and/or protocol. Such cohorts were considered as separate trials, providing 35 trials in total in six platform categories: eight for vector, eleven for mRNA, nine for protein subunit, five for inactivated, and one for DNA and virus-like particle (VLP) vaccines. The vector group had the largest total number of trial participants and was selected a reference category for the platform analysis. The funnel plot showed statistically significant asymmetry (*p* = 0.0496) with a lack of small trials with efficacy of around ≤50% (Figure 2). A full list of the included trials, characteristics of their designs, vaccines used, and participant populations are presented in Table 1.

### 3.2. Characteristics of Trials, Vaccines, and Participants, Sources of Bias and Effect Modifiers

All trials had a parallel group design and, except two, used placebo control. The COV002 and COV003 trials used a meningococcal vaccine as a control to improve blinding in terms of local post-injection reaction. Part of the trials allowed for crossover between groups and non-trial vaccinations, which could cause bias towards underestimation of vaccine efficacy. To avoid this, only trial results before crossover and non-trial vaccination were considered for the study. Except one, all vaccines were monovalent and based on the S-protein of the ancestral wild-type strain (Wuhan-Hu-1). The VAT00008 trial used a bivalent vaccine based on the ancestral and Beta strains. All vaccines were administered intramuscularly, with, predominantly, a two-dose regimen. Vaccine valency and the number of doses were prespecified by the protocol as the vaccine-level effect modifiers.

Half of the trials used FDA/CDC criteria (standard) for the definition of symptomatic COVID-19 case: presence of ≥1 systemic or respiratory symptom together with positive RT-PCR testing for SARS-CoV-2 [61,62]. Other trials required simultaneous presentation of >1 symptom and/or their longer duration (alternative). Some of the latter also used standard criteria for sensitivity analysis, which were considered for the study over alternative if corresponding results were presented. In majority of the trials, count of COVID-19 cases for the endpoint started two weeks after the last vaccine dose. COVID-19 cases were adjudicated by independent committee in one half of the trials and by observer in another. Length of period before the start of COVID-19 cases count (prespecified by the protocol), COVID-19 case criteria and adjudication method were considered for adjustment as sources of the endpoint measurement bias.

Baseline characteristics of PP and mITT populations within the single trial were similar in all cases when corresponding data were presented. On this basis, baseline values of the PP population were used for imputation of the missing baseline values of the mITT population of the same trial and vice versa, if needed for meta-regression. The mean age of trial populations differed considerably, with an average of 30.9 years. A majority of the trials were done in populations exclusively ≥18 years, with remarkable variations in the proportion of participants ≥65 years. Most of the trials had an approximately equal sex split. In terms of race, participants in the trials were predominantly White. There was a considerable variation in the proportion of participants with COVID-19-related comorbidity and a baseline seropositive status. There was also a large variation in the baseline (in control group) COVID-19 rate with a trend towards an increase with later onset of the trial clinical phase (Table 2). This is in line with changes in the dominant SARS-CoV-2 strain from less to more contagious that was observed during the pandemic.

There was evidence of the age effect on clinical efficacy for different types of vaccine platforms. Efficacy in ≥65 years was lower than in <65 years [12,27,42,49]. Likewise, vaccines investigated across all age groups showed smaller effects in participants younger than 6 years compared to those in older participants [25,33,44]. In addition, there was evidence of the viral strain effect on vaccine efficacy. Efficacy against COVID-19 cases caused by Mu and Omicron strains was lower compared to the earlier variants [16,47,49]. Similar results were available for Delta strain [55,58]. Age and strain causing COVID-19 were considered as the population-level effect modifiers. There was no evidence of the effect of serologic status on vaccine efficacy. Adjustment for the strain was complicated because not all trials presented the distribution of COVID-19 cases between SARS-CoV-2 variants. The baseline risk of the disease (in control group) was considered a reliable proxy of the strain as differences in the contagiousness of SARS-CoV-2 variants is well known. It was summarized with log-odds because rates were not reported in several trials (Table 2).

### 3.3. Analysis of Platform Type Effect in PP Set

The PP analysis comprised 35 trials. The average reduction in incidences of first symptomatic COVID-19 on vaccines in the analysis was 74% (IRR = 0.26 [0.21; 0.33], *p* < 0.001) with high and statistically significant heterogeneity (I^2^ = 81.3, *p* < 0.001).

**Figure 3:**
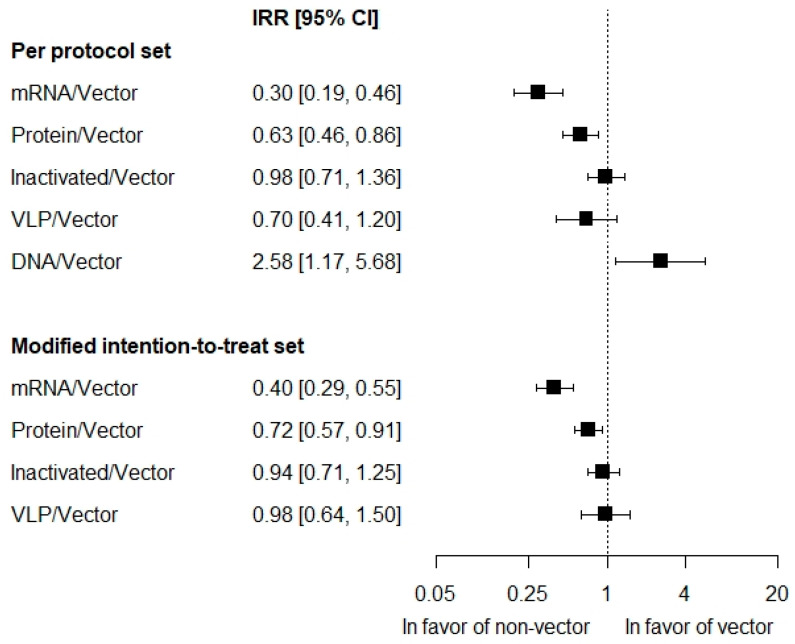
Incidence of first symptomatic COVID-19 of any severity on the vaccine platforms relatively to the vector platform.

The analysis of platform type in the PP set was adjusted for the number of vaccine doses, mean age, baseline log-odds of COVID-19, and presence of the HERALD trial (R^2^ = 95.5%). Rate of symptomatic COVID-19 on mRNA vaccines was significantly lower than the vector, by 70%: IRR = 0.30 [0.19; 0.46], *p* = 0.001 (Figure 3). The rate of cases on protein subunit vaccines was significantly lower than the vector, by 37%: IRR = 0.63 [0.46; 0.86], *p* = 0.012. There was no difference in the rate of cases for inactivated and VLP vaccines against the vector: IRR = 0.98 [0.71; 1.36], *p* = 0.913 and 0.70 [0.41; 1.20], *p* = 0.197, respectively. The rate of symptomatic COVID-19 on DNA vaccines was significantly higher than the vector, by 158%: IRR = 2.58 [1.17; 5.68], *p* = 0.034.

There was an effect of other factors on the clinical efficacy of vaccines. A higher number of vaccine doses was significantly associated with increased efficacy. One additional dose reduced the rate of symptomatic COVID-19 by 32%: IRR = 0.68 [0.52; 0.87], *p* = 0.007. Likewise, a higher mean age was significantly associated with reduction in the disease rate by 3% per one additional year: IRR = 0.97 [0.96; 0.99], *p* = 0.002. In contrast, an increase in baseline log-odds of symptomatic COVID-19 was significantly associated with decreased vaccine efficacy: IRR = 1.61 [1.36; 1,91], *p* = 0.001. Despite the abovementioned adjustments, there was a large residual heterogeneity that was not explained by any of the considered variables. This heterogeneity was found to be caused by the HERALD trial only, suggesting the presence of an unidentified effect modifier/source of bias. Adjustment for the presence of the HERALD trial increased the model R^2^ from 42.3 to 95.5%. It was significantly associated with decreased vaccine efficacy compared to the other trials: IRR = 7.03 [4.14; 12.0], *p* = 0.001. No other variables were related to heterogeneity in the trial results, and informed the model. Output of the meta-regression model is available in the supplementary materials.

### 3.4. Analysis of Platform Type Effect in mITT Set

The mITT analysis comprised 26 trials and lacked a DNA category. Average vaccine efficacy in the analysis was lower than that of the PP set: a reduction in the incidence of first symptomatic COVID-19 on vaccines was 60% (IRR = 0.40 [0.33; 0.49], *p* < 0.001) with high and statistically significant heterogeneity (I^2^ = 84.8%, *p* < 0.001).

Results of the meta-regression in the mITT set were consistent with those of the PP set. The model included adjustment for the mean age and baseline log-odds of COVID-19 (R^2^ = 87.4%). There was no adjustment for the HERALD trial due to its absence in the mITT analysis. In addition, there was no adjustment for the number of vaccine doses. This value varied among participants in mITT populations of the trials, while corresponding mean values were not reported, precluding the adjustment. Absence of this adjustment was considered to explain the slightly smaller goodness-of-fit for the model in the mITT set compared to the PP analysis (supported by similar model R^2^ [87.6%] for PP set with the number-of-doses covariate excluded). Rate of symptomatic COVID-19 on mRNA vaccines was significantly lower than on the vector by 60%: IRR = 0.40 [0.29; 0.55], *p* = 0.001 (Figure 3). The rate of the symptomatic disease on protein subunit vaccines was significantly lower than on the vector, by 28%: IRR = 0.72 [0.57; 0.91], *p* = 0.017. There was no difference for inactivated and VLP vaccines compared to the vector: IRR = 0.94 [0.71; 1.25], *p* = 0.668 and 0.98 [0.64; 1.50], *p* = 0.892, respectively.

An increase in the mean age by one year was significantly associated with an increase in vaccine efficacy by 2% (IRR = 0.98 [0.98; 0.99], *p* = 0.001), and an increase in baseline odds of COVID-19 was significantly associated with a decrease in it (IRR = 1.34 [1.13; 1.58], *p* = 0.002).

## 4. Discussion

The aim of this systematic review was to investigate the effect of platform type on the clinical efficacy of SARS-CoV-2 vaccines. For this purpose, the rates of symptomatic COVID-19 were compared between vaccine platforms using the results of available RCTs, with control for confounding and bias. The adjusted meta-regression showed the presence of systematic differences between the vaccine platforms in this regard. Rates of symptomatic COVID-19 on mRNA and protein subunit vaccines were lower than those of vector vaccines. In turn, rates of symptomatic COVID-19 on the latter were similar to those on inactivated and VLP-vaccines. In contrast, the rates of symptomatic COVID-19 on DNA vaccines were higher than those of vector vaccines. Change in the rate according to the type of platform implies interaction between these variables. Thus, the type of platform is an effect modifier of the clinical efficacy of SARS-CoV-2 vaccines. This observation supports the hypothesis about the differences in characteristics of immune response to different types of SARS-CoV-2 vaccines. Given the nature of the endpoint in the study and direction of the described relative effects of the platforms, mRNA and protein subunit vaccines are the most effective for the prevention of symptomatic COVID-19; hence, their development should be prioritized over other vaccine types. The generalizability of this conclusion is limited to prime vaccination settings and immunocompetent individuals.

The credibility of the study conclusion is determined by several aspects. It included a large number of trials, which provided power for the analysis. Power is additionally supported by the nature of the endpoint used, which attained a large number of trial-level events. The large number of trials also provided the possibility of adjustment, which is essential in a non-randomized study. Adjustment allows for a consideration of results free of confounding and bias, as RCTs represent a well-controlled environment regarding the sources of heterogeneity in their results. This is illustrated by the high goodness-of-fit of the model for both analysis sets. Restriction with eligibility criteria ensured the inclusion of trials with a low risk of selection and detection bias, while the consistency of PP and mITT analyses assured a low risk of attrition bias. The latter additionally extends the generalizability of the study results: PP analysis represents effects in ideal settings, while mITT analysis more closely resembles routine settings when full vaccination is not always possible. Differences in the clinical efficacy of the vaccine platforms were present in both situations.

The main limitations of this study are the single trials in the DNA and VLP categories, which restricted inferences regarding these platforms. Another limitation is the generally short follow-up in the included trials. It is unknown whether the demonstrated relative efficacy of the platforms persists, given the immunity waning [63]. Differences in efficacy between the platforms should be interpreted carefully as there is no established threshold of clinical importance for it. Additionally, results are applicable to the platform categories in general and do not preclude differences between individual vaccines within a single category. This is illustrated by the case of the HERALD trial, which showed a considerably smaller effect than other trials in the mRNA category. Given the similarity of the characteristics of this trial to others in the category, the only reasonable explanation for this difference was the vaccine itself used in it, which apparently differed from the other mRNA vaccines in the study in terms of immunogenicity. This is unlikely to be related to the antigen structure, as all mRNA vaccines in the study were based on the full-length pre-fusion S-protein. Thus, features of the manufacturing process may be assumed to be the cause. Elaboration of this hypothesis was beyond the scope of this study and was not possible, as details of the vaccine technologies were not reported. Regardless of the borderline results of statistical testing, the funnel plot showed an absence of small-sized trials with small vaccine effects, suggesting selection bias. We suppose this is due to the phased process of the clinical development: only vaccine candidates showing sufficient immunogenicity in early-stage trials are selected for investigations of clinical efficacy in late-stage large trials.

Results of the study allow for several additional generic conclusions to be drawn regarding clinical efficacy of SARS-CoV-2 vaccines. Meta-regression confirmed with clinical data that a multi-dose vaccination regimen is more effective than a mono-dose regimen. This is consistent with the higher immunogenicity of the former described in early vaccine trials [64]. Limited cross-immunity of strains was shown as a change in the circulating strain (relatively to the vaccine strain), proxied with the baseline COVID-19 risk, leading to a decrease in vaccine efficacy. This partially explains the smaller than expected real-life effect of the vaccination and stresses the necessity of prompt updates of SARS-CoV-2 vaccines. Finally, the effects of age on clinical efficacy were demonstrated with positive linear association. This contradicts the earlier conclusion, when data about decreased efficacy in early and old age suggested an inverse U-shaped association. The discordance may be explained by use of the mean but not categorized age for adjustment in this study. Trials in exclusively ≥65 years age group are needed to clarify details of the age effect. The linear nature of the age effect in this study means lower vaccine efficacy in early age, and was consistent with results of other studies that showed a weaker immune response to SARS-CoV-2 exposure in children [65,66]. This justifies special vaccination regimens for young individuals. For instance, more doses were used in cohorts of <5 years in the C4591007 trial: three doses were more effective than two, used as a standard in older individuals [26].

Main conclusion of this study can be elaborated with an analysis of other endpoints, in particular, asymptomatic COVID-19 and mortality. A comparison of the vaccine platforms in booster vaccination settings, which is more relevant nowadays, is also needed. Results also pose the question as to whether the discrepancy in efficacy of platforms is SARS-CoV-2-specific or generic. It can be addressed with analyses of other disease areas, where different types of vaccines are available.

## 5. Conclusions

The type of platform is an effect modifier of the clinical efficacy of SARS-CoV-2 vaccines. mRNA and protein subunit vaccines have the highest efficacy for the prevention of symptomatic COVID-19.

## Figures and Tables

**Table 1:** Included trials, characteristics of their designs, vaccines used, and participants in populations received at least one vaccine dose.

Trial Acronym or ID	Vaccine	Doses	COVID-19 Case			Start Date (dd/mm/yy) ^§^	Mean Age, y	Age Group <18/≥65, %	Men, %	White/Black/Asian, %	Sero+, %	≥1 Co-Existing Disease, %
			Criteria ^†^	Judging Method ^‡^	Count Day ^∫^							
**Vector**												
COV002 (low dose) [8,9]	ChAdOx1 nCoV-19	2	ALT	COM	15	31.05.20	42.0	0.0/7.4	40.0	92.0/0.5/5.1	-	35.9
COV002 (standard dose) [8,9]	ChAdOx1 nCoV-19	2	ALT	COM	15	09.06.20						
COV003 [8,9]	ChAdOx1 nCoV-19	2	ALT	COM	15	23.06.20	37.0	0.0/3.1	45.5	68.3/9.2/2.3	-	36.5
COV005 [10,11]	ChAdOx1 nCoV-19	2	ALT	COM	15	24.06.20	30.0	0.0/2.1	56.5	12.8/70.3/-	27.4	7.5
NCT04516746 [12,13]	ChAdOx1 nCoV-19	2	ALT	COM	15	28.08.20	51.0	0.0/22.4	55.6	79.0/8.3/4.4	2.8	60.0
ENSEMBLE [14,15]	Ad26.COV2.S	1	STD	COM	14	21.09.20	52.0	0.0/33.5	54.9	58.7/19.4/3.3	9.6	40.8
ENSEMBLE2 [16]	Ad26.COV2.S	2	STD	COM	14	16.11.20	52.0	0.0/35.9	52.6	76.4/8.2/8.7	11.1	41.4
NCT04526990 [17]	Ad5-nCoV	1	STD	COM	28	22.09.20	39.2	0.0/10.1	66.0	21.9/0.0/46.4	-	-
**mRNA**												
C4591001 [18,19,20]	BNT162b2	2	STD	OBS	7	27.07.20	49.7	1.7/20.0	50.9	82.0/9.6/4.3	3.2	44.0
C4591001 (12–15 years) [21,22]	BNT162b2	2	STD	OBS	7	15.10.20	13.6	100.0/0.0	51.0	85.5/4.8/6.3	4.1	-
C4591007 (5–11 years) [23,24]	BNT162b2	2	STD	OBS	7	07.06.21	8.2	100.0/0.0	52.1	78.9/6.5/6.0	8.7	20.5
C4591007 (2–4 years) [25,26]	BNT162b2	3	STD	OBS	7	21.06.21	3.0	100.0/0.0	49.9	79.6/4.9/7.4	13.0	12.8
C4591007 (0.5–1 years) [25,26]	BNT162b2	3	STD	OBS	7	21.06.21	1.3	100.0/0.0	49.5	78.9/3.7/7.4	7.5	4.7
COVE [27,28]	mRNA-1273	2	STD	COM	14	27.07.20	51.4	0.0/24.8	52.7	79.2/10.2/4.6	2.0	22.2
TeenCOVE [29,30,31]	mRNA-1273	2	STD	OBS	14	09.12.20	14.3	100.0/0.0	51.4	83.9/3.4/5.9	5.4	-
KidCOVE (6–11 years) [30,32]	mRNA-1273	2	STD	OBS	14	09.08.21	8.5	100.0/0.0	50.8	65.6/10.0/9.9	8.6	27.5
KidCOVE (2–5 years) [30,33]	mRNA-1273	2	STD	OBS	14	18.10.21	3.0	100.0/0.0	50.8	76.5/4.5/6.0	8.6	14.3
KidCOVE (0.5–1 years) [30,33]	mRNA-1273	2	STD	OBS	14	18.10.21	1.3	100.0/0.0	51.1	79.0/3.1/4.9	6.1	22.8
HERALD [34,35]	CVnCoV	2	STD	COM	15	11.12.20	43.0	0.0/12.7	54.8	45.5/1.9/0.3	11.9	-
**Protein subunit**												
2019nCoV-501 [36,37,38]	NVX-CoV2373	2	STD	OBS	7	17.08.20	32.0	0.0/4.2	57.4	3.5/95.3/1.2	30.2	23.0
2019nCoV-302 [39,40]	NVX-CoV2373	2	STD	OBS	7	28.09.20	55.0	0.0/27.2	51.6	94.3/0.4/3.1	4.2	44.7
PREVENT-19 [41,42]	NVX-CoV2373	2	STD	OBS	7	27.12.20	46.7	0.0/12.6	52.2	75.0/11.8/4.1	6.5	47.3
PREVENT-19 (12–17 years) [43]	NVX-CoV2373	2	STD	OBS	7	26.04.21	13.8	100.0/0.0	52.5	74.4/13.9/3.4	16.1	-
COVOVAX-Ped (12–17 years) [44,45]	SII-NVX-CoV2373	2	STD	OBS	14	--.08.21	14.3	100.0/0.0	52.6	0.0/0.0/100.0	12.8	-
COVOVAX-Ped (2–11 years) [44,45]	SII-NVX-CoV2373	2	STD	OBS	14	--.09.21	6.7	100.0/0.0	49.8	0.0/0.0/100.0	11.5	-
NCT04646590 [46]	ZF2001	3	STD	COM	7	12.12.20	36.8	0.0/6.4	67.5	0.3/0.0/81.2	0.0	13.2
SPECTRA [47]	SCB-2019	2	ALT	COM	14	24.03.21	32.1	0.0/1.4	53.1	20.2/9.9/45.5	48.5	18.1
VAT00008 [48,49]	CoV2 preS dTM-AS03	2	ALT	COM	14	19.10.21	36.1	0.0/6.0	58.4	0.6/44.3/39.7	75.0	32.2
**Inactivated**												
NCT04510207 [50]	WIV04	2	ALT	COM	14	16.07.20	36.2	0.0/2.1	84.5	-/-/-	4.9	-
NCT04510207 [50]	HB02	2	ALT	COM	14	16.07.20	36.2	0.0/2.1	84.7	-/-/-	5.0	-
PROFISCOV [51,52]	CoronaVac	2	ALT	COM	14	21.07.20	39.5	0.0/5.1	35.8	75.2/5.2/2.5	10.1	55.9
NCT04582344 [53,54]	CoronaVac	2	ALT	OBS	14	15.09.20	45.0	0.0/0.0	57.8	-/-/-	0.0	60.9
NCT04641481 [55]	BBV152	2	ALT	COM	14	16.11.20	40.1	0.0/10.9	67.1	0.0/0.0/100.0	30.4	-
**DNA**												
CTRI/2021/01/030416 [56,57]	ZyCoV-D	3	-	COM	28	16.01.21	36.5	3.4/7.5	67.1	0.0/0.0/100.0	13.4	5.2
**VLP**												
NCT04636697 [58,59,60]	CoVLP + AS03	2	STD	COM	7	15.03.21	32.8	0.0/0.5	49.1	88.8/7.0/1.2	14.8	14.4

^†^ ALT—alternative, STD—standard. ^‡^ COM—committee, OBS—observer. ^∫^ Day after the last vaccine dose when count of COIVD-19 cases for the endpoint started. ^§^ Start of clinical phase (enrollment of the first patient).

**Table 2:** Trial results.

Trial Acronym or ID	Vaccine	PP Population					mITT Population				
		Vaccine		Control		IRR [95% CI]	Vaccine		Control		IRR [95% CI]
		n/N ^†^	Rate ^‡^	n/N ^†^	Rate ^‡^		n/N ^†^	Rate ^‡^	n/N ^†^	Rate ^‡^	
**Vector**											
COV002 (low dose) [8,9]	ChAdOx1 nCoV-19	3/1367	14.9	30/1374	150.2	0.10 [0.03; 0.33]	108/10 013	-	227/9 999	-	0.47 [0.38; 0.60]
COV002 (standard dose) [8,9]	ChAdOx1 nCoV-19	15/2377	56.4	38/2430	142.4	0.40 [0.22; 0.72]					
COV003 [8,9]	ChAdOx1 nCoV-19	12/2063	56.2	33/2025	157.0	0.36 [0.19; 0.69]					
COV005 [10,11]	ChAdOx1 nCoV-19	43/935	66.3	81/960	121.1	0.55 [0.37; 0.80]	25/944	63.7	37/938	95.9	0.67 [0.38; 1.13]
NCT04516746 [12,13]	ChAdOx1 nCoV-19	141/17,617	39.2	184/8528	118.8	0.33 [0.27; 0.41]	374/21 583	59.7	370/10 797	129.3	0.46 [0.40; 0.53]
ENSEMBLE [14,15]	Ad26.COV2.S	114/19,514	36.6	345/19,544	111.4	0.33 [0.26; 0.41]	192/19 744	60.3	429/19 822	135.2	0.45 [0.37; 0.53]
ENSEMBLE2 [16]	Ad26.COV2.S	12/6024	6.9	52/5615	32.6	0.21 [0.10; 0.40]	-	-	-	-	-
NCT04526990 [17]	Ad5-nCoV	45/10,660	-	105/10,590	-	0.43 [0.30; 0.60]	169/17 899	84.2 ^¶^	336/17 878	169.7 ^¶^	0.50 [0.41; 0.60]
Pooled ^§^						0.36 [0.30; 0.42]					0.47 [0.43; 0.51]
**mRNA**											
C4591001 [18,19,20]	BNT162b2	8/17,411	3.6	162/17,511	72.9	0.05 [0.02; 0.10]	50/21 314	12.5	275/21 258	69.1	0.18 [0.13; 0.24]
C4591001 (12–15 years) [21,22]	BNT162b2	0.5/1002	3.2	16.5/973	112.2	0.03 [0.00; 0.48]	3/1 120	11.7	35/1 119	140.0	0.08 [0.02; 0.27]
C4591007 (5–11 years) [23,24]	BNT162b2	3/1273	9.3	16/637	100.6	0.09 [0.02; 0.32]	3/1 463	6.2	17/719	72.3	0.09 [0.02; 0.30]
C4591007 (2–4 years) [25,26]	BNT162b2	9/498	111.1	13/204	393.9	0.28 [0.11; 0.71]	169/2 135	214.7	121/1 058	317.6	0.68 [0.53; 0.86]
C4591007 (0.5–1 years) [25,26]	BNT162b2	4/296	95.2	8/147	400.0	0.24 [0.05; 0.90]	123/1 272	236.5	78/631	298.9	0.79 [0.59; 1.06]
COVE [27,28]	mRNA-1273	11/14,134	3.3	221/14,073	67.6	0.05 [0.02; 0.09]	26/15 181	-	276/15 170	-	0.09 [0.06; 0.14]
TeenCOVE [29,30,31]	mRNA-1273	2/2142	3.3	9/1045	32.4	0.10 [0.01; 0.49]	-	-	-	-	-
KidCOVE (6–11 years) [30,32]	mRNA-1273	3/2644	5.0	4/853	21.7	0.23 [0.03; 1.37]	-	-	-	-	-
KidCOVE (2–5 years) [30,33]	mRNA-1273	119/2594	175.0	61/858	277.0	0.63 [0.46; 0.88]	-	-	-	-	-
KidCOVE (0.5–1 years) [30,33]	mRNA-1273	51/1511	138.2	34/513	279.8	0.49 [0.31; 0.79]	-	-	-	-	-
HERALD [34,35]	CVnCoV	83/12,851	47.8	145/12,211	92.4	0.52 [0.39; 0.69]	-	-	-	-	-
Pooled ^§^						0.19 [0.10; 0.37]					0.22 [0.10; 0.53]
**Protein subunit**											
2019nCoV-501 [36,37,38]	NVX-CoV2373	51/1408	-	96/1362	-	0.51 [0.37; 0.72]	107/2 107	-	170/2 096	-	0.63 [0.50; 0.79]
2019nCoV-302 [39,40]	NVX-CoV2373	10/7020	6.5	96/7019	63.4	0.10 [0.05; 0.20]	42/7 569	18.5	141/7 570	62.6	0.30 [0.21; 0.42]
PREVENT-19 [41,42]	NVX-CoV2373	14/17,312	3.3	63/8 140	34.0	0.10 [0.05; 0.17]	121/19 714	21.2	141/9 868	51.9	0.41 [0.32; 0.52]
PREVENT-19 (12–17 years) [43]	NVX-CoV2373	6/1205	2.9	14/594	14.2	0.21 [0.08; 0.53]	11/1 484	3.0	18/748	9.9	0.30 [0.14; 0.64]
COVOVAX-Ped (12–17 years) [44,45]	SII-NVX-CoV2373	2/346	14.7 ^¶^	2/114	44.9 ^¶^	0.33 [0.05; 2.33]	3/346	19.2 ^¶^	2/114	38.9 ^¶^	0.49 [0.08; 2.94]
COVOVAX-Ped (2–11 years) [44,45]	SII-NVX-CoV2373	1/345	7.4 ^¶^	1/115	22.2 ^¶^	0.33 [0.02; 5.31]	1/345	6.4 ^¶^	1/115	19.2 ^¶^	0.33 [0.02; 5.31]
NCT04646590 [46]	ZF2001	158/12,625	-	580/12,568	-	0.24 [0.20; 0.29]	405/13 909	-	850/13 899	-	0.45 [0.40; 0.50]
SPECTRA [47]	SCB-2019	52/5935	100.5	155/5806	306.3	0.33 [0.23; 0.46]	63/12 153	58.9	185/11 983	176.9	0.33 [0.25; 0.45]
VAT00008 [48,49]	CoV2 preS dTM-AS03	15/315	312.5	22/333	449.0	0.69 [0.33; 1.39]	68/6 418	-	169/6 390	-	0.40 [0.30; 0.53]
Pooled ^§^						0.26 [0.16; 0.42]					0.41 [0.34; 0.49]
**Inactivated**											
NCT04510207 [50]	WIV04	26/12,743	12.1	95/12,737	44.7	0.27 [0.18; 0.42]	69/13 428	20.3	138/13 425	40.7	0.50 [0.37; 0.66]
NCT04510207 [50]	HB02	21/12,726	9.8	95/12,737	44.7	0.22 [0.14; 0.35]	48/13 436	14.1	138/13 425	40.7	0.35 [0.25; 0.48]
PROFISCOV [51,52]	CoronaVac	67/3637	133.0	133/3587	268.0	0.50 [0.37; 0.66]	126/6 195	-	252/6 201	-	0.49 [0.40; 0.61]
NCT04582344 [53,54]	CoronaVac	9/6559	31.7	32/3470	192.3	0.17 [0.08; 0.35]	74/6 646	94.8 ^¶^	76/3 568	196.7 ^¶^	0.48 [0.35; 0.66]
NCT04641481 [55]	BBV152	24/8 471	-	106/8502	-	0.22 [0.14; 0.35]	-	-	-	-	-
Pooled ^§^						0.27 [0.18; 0.40]					0.46 [0.40; 0.53]
**DNA**											
CTRI/2021/01/030416 [56,57]	ZyCoV-D	20/12,350	-	61/12,320	-	0.33 [0.19; 0.52]	-	-	-	-	-
**VLP**											
NCT04636697 [58,59,60]	CoVLP + AS03	32/8975	49.0	114/8033	200.0	0.24 [0.16; 0.36]	156/12 074	89.4	258/12 067	163.6	0.55 [0.45; 0.67]

^†^ Number of events/risk set. ^‡^ Events per 1000 person-years. ^§^ Unadjusted random-effect model estimate. ^¶^ Approximation from proportion. PP—per protocol, mITT—modified intention-to-treat, IRR—incidence rate ratio, CI—confidence interval, VLP—virus-like particle.

## Data Availability

All data used in the study are readily available at the references provided with the article.

## Supplementary Materials

The following supporting information can be downloaded at: https://www.mdpi.com/article/10.3390/vaccines12020130/s1, Formulae used for calculation of IRR; Table S1: Log-scale results with the uncentered covariates in the per protocol set; Table S2: Log-scale results with the uncentered covariates in the modified intention-to-treat set.
