# Supplementary material for: Effect of Platform Type on Clinical Efficacy of SARS-CoV-2 Vaccines in Prime Vaccination Settings: A Systematic Review and Meta-Regression of Randomized Controlled Trials
Source: Vaccines (Basel). 2024 Jan 26;12(2):130. doi: 10.3390/vaccines12020130 (PMC10892687; doi:10.3390/vaccines12020130)
Supplement: Supplementary file 1 [file vaccines-12-00130-s001.zip › vaccines-2816293-supplementary.pdf]

Supplementary materials

Formulae for calculation of incidence rate ratio and its confidence interval 1

Output of the meta-regression models 2

Formulae for calculation of incidence rate ratio and its confidence interval

1. Calculation of IRR from VE:

$$PE_{IRR} = 1 - \frac{VE}{100}$$

$$LCL_{IRR} = 1 - \frac{UCL_{VE}}{100}$$

$$UCL_{IRR} = 1 - \frac{LCL_{VE}}{100}$$

IRR – incidence rate ratio

VE – vaccine efficacy

PE – point estimate

LCL – lower confidence limit

UCL – upper confidence limit

2. Calculation of IRR from aggregated group-level data:

$$\lambda = \frac{E}{total\ person - time}$$

$$PE_{IRR} = \frac{\lambda_1}{\lambda_2}$$

$$LCL_{IRR} = exp\left(ln(IRR) - 1.96 \cdot \sqrt{\frac{1}{E_1} + \frac{1}{E_2}} \right)$$

$$UCL_{IRR} = exp\left(ln(IRR) + 1.96 \cdot \sqrt{\frac{1}{E_1} + \frac{1}{E_2}} \right)$$

E – events

λ – incidence rate

3. Approximation of rate from proportion:

$$\lambda_{p-y} = \frac{-ln(1-\pi)}{t}$$

π - proportion

Output of the meta-regression models

Table S1. Log-scale results with the uncentered covariates in the per protocol set.

Coefficient	Estimate	Standard error	z	p	Lower confidence limit	Upper confidence limit
Intercept	2.5795	0.4199	6.1430	0.0020	1.7565	3.4026
mRNA	-1.2084	0.2210	-5.4689	0.0010	-1.6414	-0.7753
Protein	-0.4668	0.1634	-2.8572	0.0120	-0.7871	-0.1466
Inactivated	-0.0204	0.1674	-0.1219	0.9130	-0.3484	0.3077
DNA	0.9482	0.4023	2.3569	0.0340	0.1597	1.7368
VLP	-0.3591	0.2762	-1.3003	0.1970	-0.9004	0.1822
Doses	-0.3918	0.1314	-2.9816	0.0070	-0.6494	-0.1343
Mean age	-0.0255	0.0055	-4.5951	0.0020	-0.0363	-0.0146
Baseline log-odds	0.4752	0.0872	5.4491	0.0010	0.3043	0.6461
Herald	1.9500	0.2706	7.2061	0.0010	1.4197	2.4804

Table S2. Log-scale results with the uncentered covariates in the modified intention-to-treat set.

Coefficient	Estimate	Standard error	z	p	Lower confidence limit	Upper confidence limit
Intercept	1.0418	0.2653	3.9275	0.0240	0.5219	1.5617
mRNA	-0.9247	0.1644	-5.6235	0.0010	-1.2470	-0.6024
Protein	-0.3312	0.1222	-2.7102	0.0170	-0.5708	-0.0917
Inactivated	-0.0618	0.1462	-0.4225	0.6680	-0.3484	0.2248
VLP	-0.0190	0.2171	-0.0873	0.8920	-0.4445	0.4066
Mean age	-0.0156	0.0048	-3.2565	0.0010	-0.0251	-0.0062
Baseline log-odds	0.2915	0.0848	3.4397	0.0020	0.1254	0.4577
